# The potential recording of North Ionian Gyre’s reversals as a decadal signal in sea level during the instrumental period

**DOI:** 10.1038/s41598-024-55579-4

**Published:** 2024-02-28

**Authors:** Matteo Meli

**Affiliations:** Department of Biological, Geological and Environmental Sciences, University of Bologna, 40126 Bologna, Italy

**Keywords:** Physical oceanography, Planetary science

## Abstract

In recent decades, the north Ionian Sea, central Mediterranean Sea, has witnessed shifts in surface current circulation from cyclonic to anticyclonic and vice versa at the quasi-decadal scale, a phenomenon termed the North Ionian Gyre (NIG) reversal. This process impacts parameters such as sea level by altering thermohaline properties and redistributing water masses at the sub-basin scale. Previous studies have shown that during anticyclonic (cyclonic) phases, the Ionian sea-level trend is falling (rising), the opposite of what is observed in other Mediterranean sub-basins. Assuming that sea level records reversals, this study employed signal decomposition to analyze satellite altimetry data and tide gauge observations across the region, some dating back to the 1900s. A distinct quasi-decadal periodicity emerges as the second dominant oscillatory mode from all independent observations, aligning its peaks and troughs (i.e., changes in sea-level trend) with known NIG reversals and associated changes in the state of North Ionian vorticity. Furthermore, this mode from altimetry data clearly depicts the spatial variability of sea level attributed to the opposite NIG states. This periodicity appears coherent among different sub-basins within the domain, especially in the eastern Mediterranean, with a shared generalized out of phase and weakening occurred from the 1930s to the 1950s. This study presents a century-scale reconstruction of NIG reversals, contributing to the understanding of this phenomenon prior to 1987 using direct observations from sea-level data.

## Introduction

**Figure 1:**
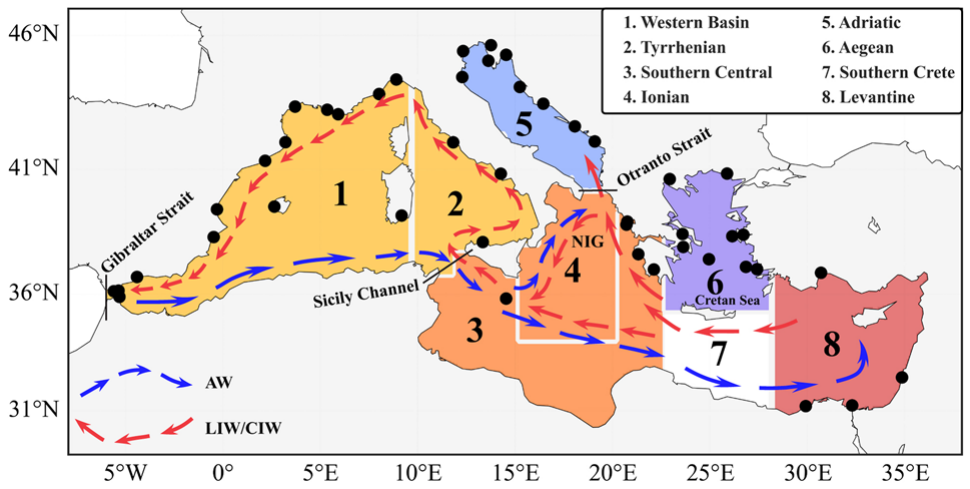
Locations of tide gauges used in this study (black dots) superimposed on the Mediterranean Sea sub-basins (identified by numbers). Sub-basins represented by the same color (i.e., 1 + 2 and 3 + 4) were combined in the analysis and treated as a single sub-basin. Blue and red arrows are representative of the main, simplified path of AW and LIW/CIW, respectively. The figure was produced using the Basemap package (version 1.4.0) in Python 3.11.5 (https://matplotlib.org/basemap/stable/).

The Mediterranean Sea (please refer to Fig. 1 for the location of names mentioned in the text) has been identified as a key climate hot spot^1^ and holds unique significance despite encompassing just around 0.3% of the global ocean’s total volume. Similar to the global ocean but on reduced temporal and spatial scales, the Mediterranean Sea showcases unique oceanographic processes^2^ and a complex circulation pattern^3^, including a specific basin-scale conveyor belt. The latter refers to a basin-wide circulation driven by thermohaline contrasts between the inflow of the relatively low saline Atlantic Water (AW) from the Strait of Gibraltar, confined to the upper 200 m layer^3^, and the intermediate waters formed in both the Levantine Basin (LIW) and Cretan Sea (CIW). In essence, LIW/CIW is AW transformed into a saltier and warmer water mass during its eastward journey, which then cyclonically recirculate westward at an intermediate depth (with an average depth between 200 and 400 m)^4,5^ and eventually exiting into the Atlantic Ocean via Gibraltar^3^. These water masses are the only ones ubiquitous throughout the entire domain^6^, as the advection of deeper masses is blocked by the Sicily Channel (max depth around 315 m), which separates the Western (WMed) and Eastern (EMed) Mediterranean Sea.

The comprehension of Mediterranean oceanography has undergone significant transformation over the last decades, shifting from a static perspective to an intensely dynamic view. Indeed, circulation patterns and thermohaline properties in the Mediterranean Sea are known nowadays to be not steady over time but are subject to changes, as observed in events such as the Eastern Mediterranean Transient (EMT)^7^ and the Adriatic–Ionian Bimodal Oscillating System (BiOS)^8^. The EMT, a climatic event occurred between the late 1980s and early 2000s^9^, involved complex atmospheric, oceanic, and hydrological interactions that shifted the EMed deep-water formation from the southern Adriatic to the Cretan Sea^10^. The BiOS, instead, represents a feedback mechanism that links the EMed’s deep thermohaline cell, originating in the southern Adriatic Sea, to the surface circulation of the North Ionian Gyre (NIG)^11^. This connection induces a quasi-decadal shift in circulation, from anticyclonic to cyclonic and vice versa, resulting in a fluctuating widespread modification of thermohaline properties and water mass redistribution in the Mediterranean. In detail, changes in the NIG state affect the path of AW towards the Levantine Basin^12^: during cyclonic phases, there is a direct short route, whereas during anticyclonic phases, there is a strong deflection towards the northeast, with the AW partially entering the Adriatic Sea through the Otranto Strait^13^, and impacting several variables such as the properties of dense water that forms in the Adriatic region^14^. Depending on the length of the AW path in these configurations, the thermohaline properties of the EMed change. This is because the relatively fresher flow of AW is either strongly reduced or enhanced when reaching the area of intermediate waters formation^15^.

The quasi-decadal NIG surface circulation reversal episodes has become of fundamental importance in the study of oceanography and other fields related to the Mediterranean Sea. Occurrences of reversals have been documented since the late 1980s, based on modeling, experimental studies and direct observations. These reversals have transitioned from a cyclonic to an anticyclonic state around the years 1987, 1998, 2010, and 2019, and vice versa around 1991, 2006, and 2016 (see^6,16–19^ and references therein). A real periodicity of this phenomenon, however, remains uncertain^3^. NIG reversal also impacts the sea level^20,21^, altering the redistribution of mass and thermohaline properties^8,11,22^. Thus, occurrence of NIG reversals may inevitably be recorded by the sea surface height (a concept already termed ‘Liquid Climate Archives’^23^) within its complex evolution, particularly linked to the alteration of the sterodynamic component^24^ of the sea level. The effect of reversal episodes on sea level has also been observed in the altimetric signal over the past three decades, as hypothesized by Refs.^18,20,25^; this led to the so-called ‘breathing oscillation’^26^ in sea level throughout the domain, i.e., a rising (falling) sea-level trend in the Ionian Sea and a simultaneous falling (rising) trend in the other Mediterranean sub-basins during cyclonic (anticyclonic) phases. This phenomenon has been attributed to the effects of the NIG reversals on changes in the sterodynamic component, controlling the short-term trends in sea level within the Mediterranean Sea^21^.

Despite numerous studies, different factors or triggers have been considered as responsible for the reversal phenomenon (see^19^ for a comprehensive review), mainly related to internal dynamics^8,27,28^ or wind forcing^29,30^. However, no formal conclusion has been reached yet. It is therefore essential to understand the evolution of NIG inversions over time more thoroughly, as they are considered a direct indicator of the Mediterranean Sea’s variability^31^ and may be pivotal for projections under various climate change scenarios. Currently, only a few studies have addressed the potential reconstruction of NIG reversals over the past century, based on tidal records^23^, regional ocean circulation models^32^ and historical observations of the occurrence of various fish species and plankton taxa in the Adriatic Sea^19^. These results only partially agree, highlighting the need for further research in this direction. To complement previous studies, a set of 46 sea level time series from tide gauges (TGs), distributed throughout the Mediterranean Sea (Fig. 1), were analyzed in this study. TGs, despite the signal’s contamination from various processes, can provide sea-level observations since the nineteen century (earlier in some circumstances) permitting long-term reconstructions. In addition, satellite altimetry (SA) data have been used to illustrate the spatial variability of sea-level changes in recent decades. The time series from both datasets were decomposed using the Ensemble Empirical Mode Decomposition (EEMD), which divides the signals into a finite number of Intrinsic Mode Functions (IMFs). These IMFs represent both oscillatory modes and residual signals, commonly referred to as the trend. Given the primary focus of this study, i.e., detecting a potential signature in the sea-level signal linked to NIG reversal episodes at the sub-basin/regional scale, a particular emphasis was placed on the decadal timescale variability, which emerged as the second mode of variability within all datasets considered.

## Results

**Figure 2:**
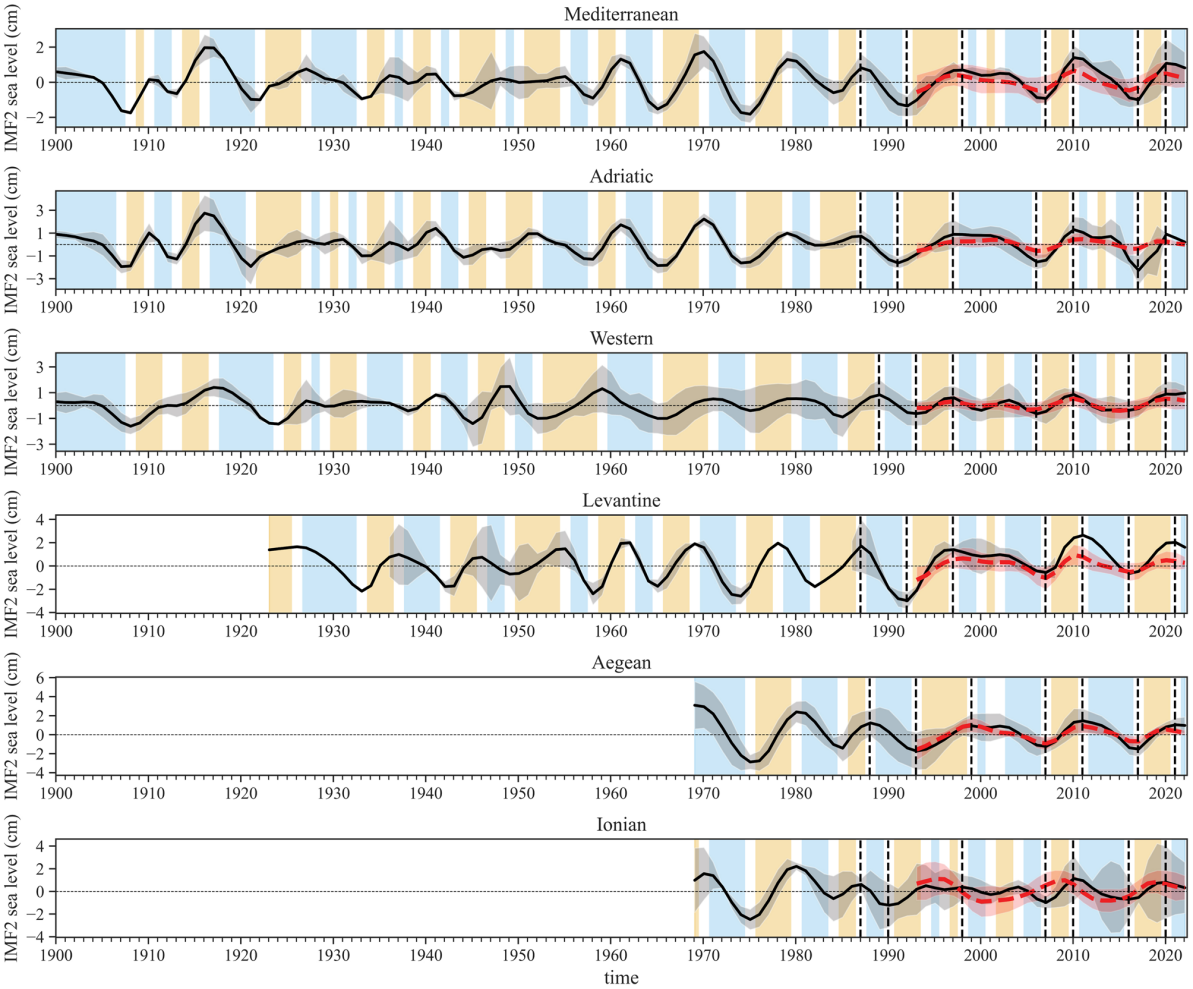
The ensemble mean of the second IMF at the sub-basin scale, computed from TGs, is represented by the solid black line, accompanied by the ± 1$\sigma$ range (shaded in gray). The red hatched lines depict the basin average mean of the second IMF derived from the altimetry data, whereas the vertical black hatched lines indicate occurrences of NIG reversal episodes documented in literature^6,18,19^. The cyan and orange sectors respectively symbolize the likely periods of the cyclonic and anticyclonic patterns of Ionian circulation. The patterns displayed from 1987 onwards match documented observations in the literature, while those depicted prior to 1987 are hypothesized in this study. Differences in the temporal extent of phases and related alignment of known reversals among different sub-basins might be attributed to the estimated uncertainty of datasets with yearly resolution (about ± 1 year), the spatially uneven distribution and length of datasets, and/or the movement of the signal across the region.

**Figure 3:**
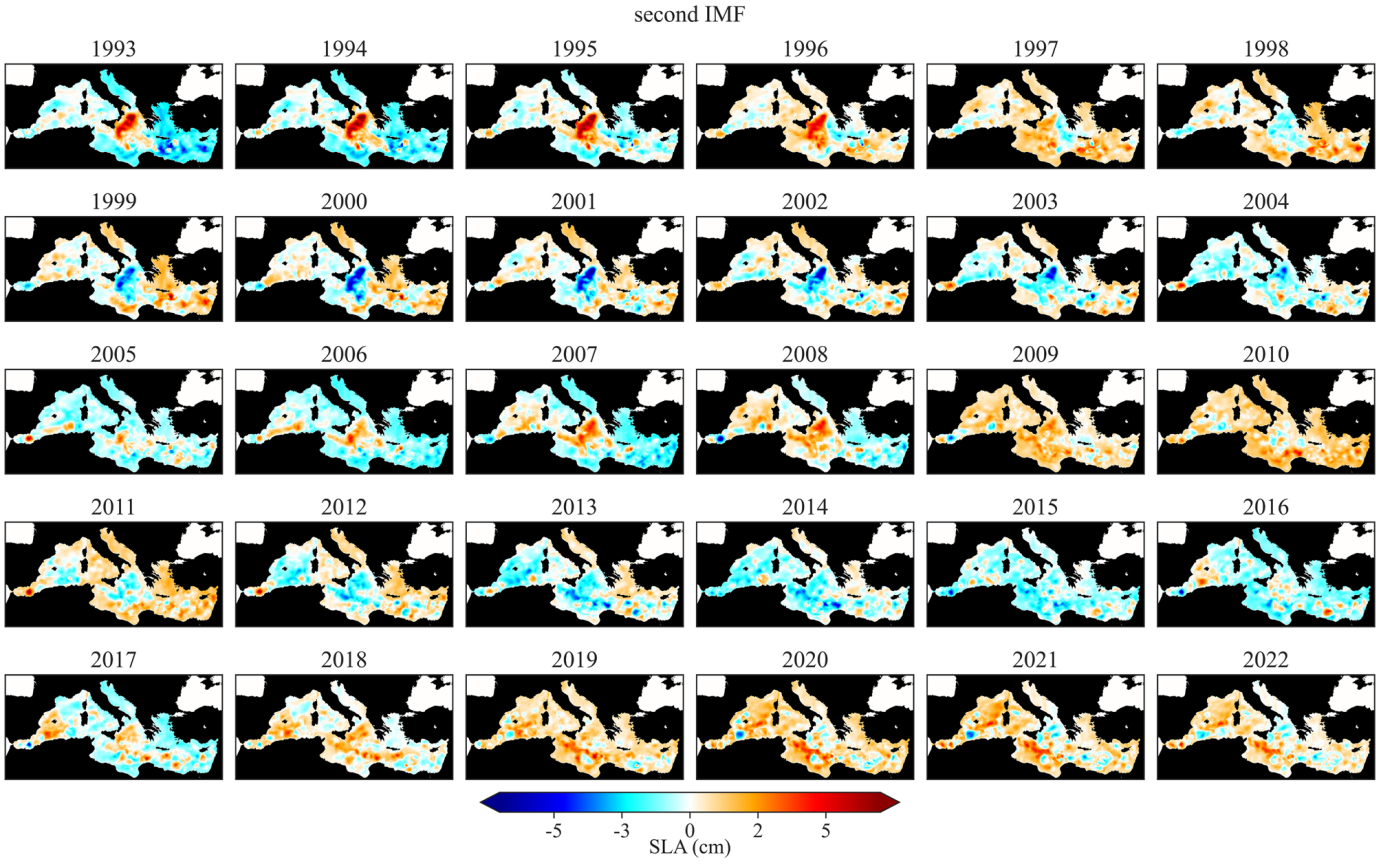
Annual mean value of sea level, associated with the second IMF, extracted from the signal decomposition using EEMD at each altimetry grid point. The ‘breathing oscillation’ in sea level, i.e. the opposite behaviour of the Ionian with respect to the other Mediterranean sub-basins, linked to the effect of the NIG reversal, is well depicted by this mode.

The second IMFs obtained from the decomposition of both TGs and SA data reveal a quasi-decadal oscillatory mode in sea level throughout the Mediterranean Sea and associated sub-basins (Fig. 2), identifying this signal as the potential focal point of this study (see the Supplementary Information for other IMFs). This oscillation, between positive and negative values, is centered around zero as a consequence of the decomposition process (see “Methods” section). This reflects the balanced nature of the fluctuations in the original signal, with each periodic signal extracted oscillating around its respective mean, which, in this case, is zero. The periods subsequent to the signal’s minima (maxima) therefore represent actual inflections in the sea-level trend, i.e., a shift from a falling (rising) to a rising (falling) trend at the quasi-decadal time scale. In terms of sea-level components (see also Supplementary Information), these shifts can be largely attributed to changes in sterodynamic properties. This refers to the variations in thermohaline properties and water mass redistribution that are the principal effects induced by NIG reversals. The occurrence of known reversal episodes, once aligned with the second IMFs, reveals a high concordance with main inflections over the last 35 years. Furthermore, the behavior of the signal, especially in the Adriatic, Aegean, and Levantine, is consistent with observations reported in the literature^21^, i.e., a generalized shift to a rising (falling) trend in sea level at the onset of anticyclonic (cyclonic) NIG states. Consequently, based on the match of these shifts, the periods following the signal’s minima (maxima) are interpreted as the anticyclonic (cyclonic) phases of the NIG throughout the time series (Fig. 2). This interpretation is based on the assumption that the relationship observed over the last 35 years extends to earlier periods in the time series, suggesting a potential correlation between these shifts and NIG phases over time. Moreover, the sea-level spatial variability of the second IMF over the last 30 years (Fig. 3), achieved from the decomposition process applied to SA data, provides further indication regarding the hypothetical correlation between this oscillatory mode and NIG reversal occurrences. Indeed, the spatial pattern of sea-level rise and fall (breathing oscillation driven by NIG reversals, discussed in Introduction) is evident across the region.

In a detailed examination of the phases shown in Fig. 2 spanning the entire Mediterranean Sea, over the 122-year period under study, cyclonic periods account for approximately 42.6% of the total. Meanwhile, anticyclonic and transitional periods represent 32.8% and 24.6%, respectively. Most of the maxima/minima in the signals align closely, typically within a $\pm 1$ year window (Table 1). This might mean that, at the yearly resolution, each reversal linked to a sea-level trend inflection can be detected within an uncertainty of at least 3 years. This is also connected to the number and length of observations available across different sub-basins. Furthermore, the observed differences in the temporal extent of potential NIG phases and occurrence of inflections at the sub-basin scale (Fig. 2) might be attributed to the movement of this signal across the sub-basins. However, a different approach and at least monthly resolution datasets are needed to account for this analysis. Due to this, the timing of potential reversals obtained from this reconstruction should be considered as a best approximation. Despite the oscillatory signal appears coherent among the sub-basins, this coherence diminishes in the period roughly between the 1930s and 1950s. During this time, in fact, a different behavior in the signal is noticeable, mostly characterized by uncertain and frequent shifts, and sometimes opposite phases between the sub-basins. The periodicity of the second IMF signals from TGs in the Mediterranean Sea has been averaging around $12.0 \pm 4.8$ years since 1900, in agreement with^18^. The spatial distribution of the second IMFs extracted from SA, illustrated in Fig. 3, underscores the variability of the second IMFs signal, with a dominant periodicity of $10.3 \pm 2.8$ years that remains consistent within the 1$\sigma$ range when compared to the periodicity observed in the TGs signals. Together with this, the average amplitude of the signal (peak to peak) in the Mediterranean Sea is $3.1 \pm 2.3$ cm.

The ensemble time series of the second IMF from both TGs and SA are coherent in terms of magnitude and phase (Fig. 2). This suggests that the relative sea-level signals from TGs accurately reflect the sea level from SA when considering a sub-basin average and an oscillatory mode, as the contamination from vertical land movements (VLMs) remains in the residuals after the decomposition (see “Methods” Section). The sole divergence noted between the two signals concerns the Ionian Sea. This lack of coherence, spatially observable in Fig. 3, is attributed to processes occurring in the central part of the Ionian where no TGs are present. These processes result in a sea-level behavior in the central region that contrasts with the basin’s edges, hence only partially captured by TGs, which are unevenly distributed around the sub-basin. It is worth noting that, as part of the NIG reversal influences on sea level might be lost within other IMFs, the entire magnitude of the phenomenon may not be captured by the second IMF alone. Indeed, accurately quantifying the consequences of reversals on sea level in terms of magnitude is beyond the scope of this study, as it requires a detailed analysis of all sea-level components at a regional-to-local scale, which is currently only partially done by Ref.^21^.

**Figure 4:**
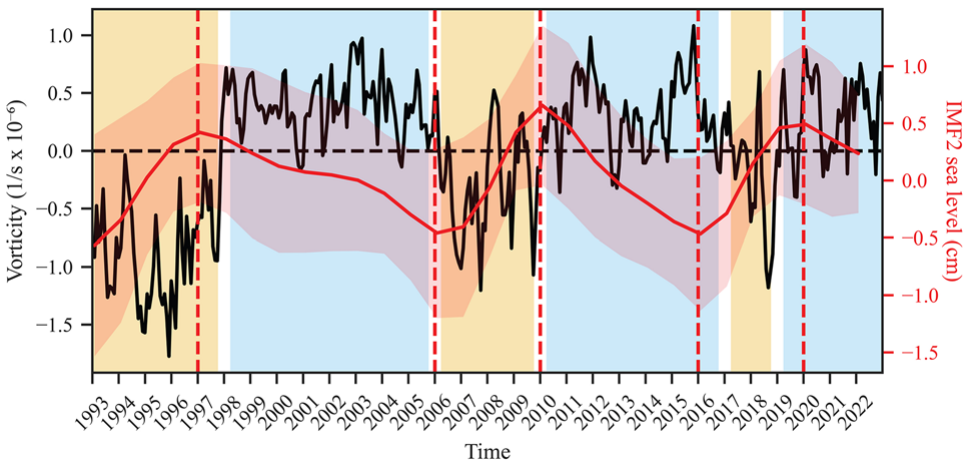
The monthly vorticity field of NIG circulation (black solid line), computed over the area 17–19.5^∘^E and 37–39^∘^N (see also “Methods” for details), where cyclonic (cyan) and anticyclonic (orange) phases are observed with positive and negative values, respectively. The second IMF averaged over the Mediterranean Sea extracted from yearly SA (red solid line), and related 1$\sigma$ (red shaded area), matches the change of state of NIG circulation accounting for maxima and minima (red hatched lines).

To further examine the properties of the second IMF in relation to the reversals, the Mediterranean average signal was compared with the vorticity time series of the NIG area over the period 1993–2021 (Fig. 4). A peculiar correlation emerges from the direct comparison, where the maxima and minima of the second mode of sea level roughly coincide with the shifts in vorticity states, the values of which are positive (negative) during cyclonic (anticyclonic) phases^17^. As stated above, an uncertainty of at least ± 1 year should always be considered to account for the spatial variability of this phenomenon in sea level^21^. Specifically, the maximum inflections in 1998, 2010, and 2019 within the SA correspond to the shift in vorticity from negative to positive values, signifying a transition from an anticyclonic to a cyclonic state. The same pattern is noted for the inverse shifts that took place in 2006 and 2016. It should be emphasized that the two signals compared in Fig. 4 represent distinct processes, each with its own evolution. Specifically, the vorticity signal is characterized by a lack of trends, typically exhibiting a pseudo positive (negative) steady-state over cyclonic (anticyclonic) phases, with occasional exceptions (e.g., the brief inversion in 2012), transitioning through rapid jumps. Thus, the correlation indicates that once a specific NIG vorticity state is established, an inflection within the sea level’s quasi-decadal mode is observed, leading to a generalized rising (falling) trend over the entire duration of the anticyclonic (cyclonic) phase.

**Table 1:** The minimum and maximum dates correspond to the peak years identified in the second IMF signals from TGs for the Mediterranean Sea and its related sub-basins. These dates potentially indicate shifts to anticyclonic (min) and cyclonic (max) states of the NIG. Since the occurrences of reversals cannot be linked to an exact moment in time and space, the year of reversal in the Mediterranean ensemble is identified as the average across sub-basins , accompanied by its uncertainty (enclosed in parentheses) which is sufficient to encompass the occurrence of the same reversal across all sub-basins.

Domain	Min dates (±)	Max dates (±)
Mediterranean	1908 (1); 1913 (0); 1922 (2); 1933 (0); 1938 (0); 1943 (2); 1950 (2); 1958 (0); 1965 (0); 1975 (1); 1984 (2); 1992 (2); 2000 (1); 2007 (1); 2017 (1)	1910 (2); 1916 (1); 1927 (1); 1936 (1); 1941 (0); 1948 (2); 1955 (2); 1961 (2); 1970 (1); 1979 (1); 1987 (2); 1998 (1); 2002 (2); 2010 (1); 2020 (1)
Adriatic	1907; 1913; 1921; 1929, 1933; 1938; 1944; 1948; 1958; 1965; 1974; 1982; 1991; 2006; 2012; 2017	1910; 1916; 1927; 1931; 1936; 1941; 1947; 1952; 1961; 1970; 1979; 1987; 1997; 2010; 2014; 2020
Western	1908; 1913; 1924; 1929; 1938; 1945; 1952; 1965; 1975; 1985; 1993; 2000; 2006; 2013, 2016	1912; 1917; 1927; 1933; 1941; 1949; 1959; 1971; 1980; 1989; 1997; 2003; 2010; 2015, 2020
Levantine	1933; 1942; 1949; 1958; 1965; 1974; 1982; 1992; 2000; 2007; 2016	1926; 1937; 1946; 1955; 1962; 1969; 1978; 1987; 1997; 2002; 2011; 2021
Aegean	1975; 1985; 1993; 2001, 2007; 2017	1980; 1988; 1999; 2002, 2011; 2021
Ionian	1975; 1984; 1990; 1996; 2001; 2007; 2016	1970; 1980; 1987; 1994; 1998; 2004; 2010; 2020

## Discussion

For the initial 22 years of the analyzed TG time series, the reconstruction is based solely on data from the Adriatic and Western regions, as no other data are available for the entire domain. According to this reconstruction, a cyclonic phase was in progress in the early 1900s, persisting until approximately 1908, when it transitioned to a short-lived anticyclonic phase lasting until around 1910. The transition to another anticyclonic state then occurred around 1913 and again in 1922, whereas a shift to a cyclonic state is observed around 1916. Generally, fluctuations observed over this period align with those reported by Ref.^23^ from TG data, which the authors interpret as the effect of either a paleo-EMT event (see also^33^) or episodes of BiOS variability, as also speculated in this study. The onset of the cyclonic phase between 1910–1913 aligns with the increase in salinity observed in the Adriatic by Ref.^34^, who referred to this event as the ‘Najade-Ciclope Ingression’. Indeed, during cyclonic periods, the relatively fresher Atlantic Water (AW) is directly deflected towards the Levantine while the saltier LIW/CIW is advected into the Adriatic Sea^35^. This leads to a relative salinization of the Adriatic. In contrast, during anticyclonic phases, the salinity in the Adriatic tends to decrease due to the direct diversion of AW northeastward by the NIG, subsequently entering the Adriatic through the Otranto Strait (see also Fig. S3). This has implications for sea level, resulting in a fall (rise) associated with an increase (decrease) in salinity, that is, alterations in the halosteric term of the steric component. This effect however, as noted by Ref.^21^, accounts for only a portion of the changes in the Adriatic sea level (roughly half and variable in time), as it also seems influenced by non-steric effects such as the pure dynamic redistribution of water mass. The latter might have a substantial influence among the sea-level components of the Adriatic, as also previously stated by Ref.^36^, who suggested that the southern Adriatic vorticity increases after the onset of an anticyclonic state of the NIG.

The period between the transitions to cyclonic (1928) and anticyclonic (1958) states presents challenges for interpretation, due to inconsistencies among the available data, namely from the Adriatic, Western, and Levantine sub-basins. The oscillatory mode for the Western seems to be out of phase; this signal, however, may not reliably depict NIG reversal episodes, as the sea level there is influenced by various sub-basin scale processes. Specifically, in the WMed, the breathing oscillation is either significantly altered or absent during the altimetry era^21^. A further challenge in interpretation arises from the Levantine curves: over this time span, their fluctuations are sometimes out of phase as well, and the limited data available exhibit strong discrepancies within the 1$\sigma$ interval. For the Adriatic, the signal over these 30 years is characterized by high-frequency fluctuations, likely providing an indication of a period characterized by short and not well-defined NIG inversions. Interestingly, Ref.^34^ documented three additional events of anomalous salinity increases around 1930 (Bois ingression), 1939 (Velirat), and 1948–1949 (Hvar), that closely align with the potential onset of brief cyclonic phases. Moreover, this period appears to be predominantly marked by an anticyclonic state, aligning with^34^ observation of ‘normal’ salinity conditions between ingressions. Considering all reconstructions, potential NIG reversals during this 30-year span are identified as transitions from cyclonic to anticyclonic states around 1933, 1938, 1943, and 1950, and vice versa around 1936, 1941, 1948, and 1955. It should be remarked, however, that fluctuations over this period are associated with large discrepancies within the 1$\sigma$ range (Fig. 2). From the 1960s onwards, the situation appears to shift drastically. All oscillatory modes, along with those from the Aegean and Ionian starting in 1969, align and remain in phase. Transitions to cyclonic conditions are evident around 1961, 1970, and 1979, while transitions to anticyclonic conditions occur around 1965, 1975, and 1984.

Since the late 1980s, the known reversals documented in the literature roughly align consistently across all sub-basins, except in a few instances linked to short events that are often (but not always) confined to a single sub-basin. This is the case with the reversal observed in the Adriatic around 2012. This brief inversion has been previously attributed to the effect of the particularly cold winter that year in the Adriatic^37^, and is also depicted in the NIG vorticity (Fig. 4), where the signal briefly changes its polarity within 2012. A similar observation is made for vorticity around 2000, together with second IMF signals within the Aegean, Ionian, Levantine, and Western sub-basins. Short-events like these are likely present throughout the signal and, as observed over the SA period, effectively contaminate the NIG state, changing its polarity but not being sufficiently strong to trigger a proper reversal. Therefore, these brief events are not considered anomalies, as they are adequately captured by the quasi-decadal signal. Although they contaminate it, these events might be helpful in fully understanding the complex history of NIG reversals. However, despite the similar, dominant quasi-decadal signal arising from all time series, the variability within sub-basins should not be neglected. As stated in the previous paragraph, data from the WMed, despite their volume and length, might not fully represent the history of NIG reversals. A different situation applies to the Levantine, which is underrepresented due to data scarcity, and to the Aegean, where observations only begin in 1969. The Ionian, being the epicenter of the reversals and underrepresented by in-situ data, exhibits significant intra-basinal variability, leading to a lack of clarity in its signal (see also Supplementary Information). Consequently, along with the Mediterranean average, the signal from the Adriatic emerges as the best proxy for reconstructing NIG reversals (as also stated by Ref.^14^), owing to its Mediterranean location and the availability of extensive, consistent long-term data series that align well within the 1$\sigma$ interval.

While the investigation of the causes behind the NIG reversal is beyond the scope of this work, discussing the potential influence and connection of the North Atlantic Oscillation (NAO^38^) with the observed decadal mode is necessary. The NAO represents the most prominent mode of winter climate variability in the Northern Hemisphere, and it affects the Mediterranean sea level, as observed in several studies (see e.g. Refs.^39–44^), through various mechanisms at scales ranging from inter-annual to multi-decadal. Overall, the NAO plays a role in determining the wind patterns around the Strait of Gibraltar, impacting the exchange of water masses between the Atlantic and the Mediterranean^40–42^, while also influencing sea-level pressure, freshwater and buoyancy flows, and riverine inputs within the Mediterranean Sea^45,46^. When applying the same signal decomposition approach to the NAO, a significant anti-correlation with the Mediterranean-averaged second IMF emerges, suggesting that a potential link cannot be excluded. Therefore, albeit with caution, it can be argued that the NAO might drive the quasi-decadal oscillation observed in regional sea level, either directly or indirectly as a forcing mechanism behind the NIG reversals. In particular, the quasi-decadal mode observed in the NAO appears to precede the one observed in sea level in the Mediterranean by 1 to 2 years, suggesting a causal relationship. Additionally, during the 1930s-1950s period, similar to sea level, the NAO’s oscillatory mode decreases in amplitude, and a general loss of distinct oscillatory patterns is observed. Moreover, during these years, the two signals are in phase, in contrast with the anti-correlation observed in the rest of the time series, indicating a potential loss of connection. This aligns with the observation that the sea level’s second IMF during this period disagrees across different sub-basins. A direct link between the NAO and sea level in the Mediterranean at a quasi-decadal scale is challenging to support for several reasons: (1) A direct mechanical surface forcing induced by the inverse barometer (IB, see also “Methods”) effect and geostrophic winds would hardly explain the spatial behavior shown in Fig. 3 (already associated with the effect of NIG reversals), which can instead be explained in terms of baroclinic response; (2) The main influence of large climate circulation patterns on sea level predominantly occurs at the multi-decadal scale (see also^40^) and has a limited effect on quasi-decadal variability^20^; (3) Wind forcing has been found to have a negligible anti-correlation with NIG reversal episodes^27^, and this would hardly support the fit between reversal episodes and the consequent sterodynamic oscillations of sea level (Fig. 2); (4) At these time scales, sea level changes within the Mediterranean Sea, linked to the coupled interaction between the NAO and the ocean, should respond uniformly as a barotropic signal transferred from the Atlantic through Gibraltar^40^, which is different from what has been observed in this study. Therefore, although additional confirmatory analyses are crucial to determine the potential cause or contributing factors of this phenomenon, it is plausible that the NAO might indirectly trigger the NIG reversal by altering parameters (e.g., rainfall, river discharge) that can modify thermohaline properties in key areas, as previously suggested by Refs.^23,46^. These authors have indeed found that variations associated with the NAO (as well as other climatic modes) interact with internal ocean dynamics in the Mediterranean, impacting not just surface levels but also the intermediate and deep circulation within the Mediterranean Sea. This could also support the hypothesis of the general mechanism of the BiOS.

It can be argued that a co-causal relationship between NIG reversals and inflections in the quasi-decadal sea-level trend would not manifest synchronously but would instead necessitate the presence of a lag time in the response. Reversals are typically associated with a specific period as the best approximation for the change in polarity of the NIG vorticity, a detail that, however, is overshadowed when data are averaged on an annual resolution. Concurrently, the information on quasi-decadal sea level, hypothesized to be connected with reversals, is approximated to the peak maximum/minimum of the inflections in the signal; however, as observable in Figs. 2 and 4, this is a gradual phenomenon, and it is not certain that the actual reversal timing can be strictly attributed to peaks. Moreover, averaging on a sub-basin scale removes the information related to the spatial propagation of the signal, thereby reducing the ability to accurately account for the timing of reversals. Furthermore, as highlighted in the Results section and by Ref.^21^, the occurrence of inflections should always be considered within a range of at least 3 years, which does not substantially differ from the temporal lags observed by Refs.^47,48^ regarding the influence of reversals on various parameters in the Adriatic. It should be noted that variabilities at the intra-annual/annual scale, induced by NIG reversals, have been observed as well by Ref.^47^, while Ref.^37^ documented the rapid transition related the brief event that occurred in 2012. Furthermore, Ref.^36^ has documented a correlation time lag of 14 months between modifications in the geostrophic current vorticities of the NIG area and those in the southern Adriatic. This provides further evidence that rapid responses in sea level to changes in NIG states might also be driven by the dynamic component (see also^21^). At last, sea level represents the integrated surface response of all processes occurring within the water column, and this may not necessarily fully and directly reflect a variation observed in a specific parameter at a particular point in space and depth.

## Conclusions

In this study, sea-level signals from TG and SA over the Mediterranean Sea were first decomposed into various dominant modes. Subsequently, a shared quasi-decadal oscillation observed across all datasets was identified and interpreted as the potential consequence related to NIG reversals. Given that some TG data have been operational for over a century, it has been possible to reconstruct the occurrences of reversals since 1900 based on observations. The reconstructions over different sub-basins are marked by good synchronicity, particularly in the EMed, with the exception of the period from 1930s to 1950s, during which the available data are either out of phase or exhibit a high-frequency variability. This is among the first attempts to reconstruct the behavior of the NIG reversal on a centennial scale. Trying to reconstruct the temporal evolution of NIG reversal episodes is of fundamental importance for a more comprehensive understanding of sea level nature, among other things, at the Mediterranean Sea scale. Although the effect of reversals on sea level appears to be an oscillatory mode, and therefore potentially inconsequential in terms of future projections, it can essentially govern the trend in the short term. It should be noted, however, that this statement might not be entirely accurate if reversals are indirectly triggered by changes in parameters more closely related to the effects of climate change. Regarding the amplitude of the signal, short-term variability of approximately 3/4 cm, though it might seem negligible, can lead to a shoreline retreat of more than a meter in regions with low-lying coasts. This has substantial implications, particularly during storm surges where the higher average sea surface in late anticyclonic (and early cyclonic) phases might exacerbate erosive conditions and reduce the efficacy of coastal defense measures. Further research is required to investigate in depth the potential connection between this oscillatory mode in sea level and NIG reversal episodes. This will be crucial to corroborate, refute, or refine this historical reconstruction, which could be invaluable in better understanding the origins of the phenomenon.

## Methods

### Tide-gauge data

The study examined a collection of 46 monthly TG records, spanning from the 1870s to 2022. Of these, 43 were sourced from the revised local reference dataset provided by the Permanent Service for Mean Sea Level^49^. Data for the TGs in Venice, Porto Corsini, and Antalya, on the other hand, were specifically retrieved from Refs.^50–52^. A detailed list can be found in the Supplementary Information (Table S1).

Observations flagged for quality issues were removed. Months needed a minimum of 15 days of observations and years required at least nine months of data to be included in the analysis. To fill gaps and extend the time span, TG datasets Genova, Malaga, Tarifa, and Palma de Mallorca were merged with observations from nearby stations (i.e., Genova II, Malaga II, Tarifa 2, and Palma de Mallorca 2) following an overlapping comparison. The effects of local wind and changes in sea-level pressure were accounted for using an IB correction; sea-level pressure fields from the ERA-20C^53^ and ERA5^54^ reanalyses for the periods 1900–1939 and 1940–2022, respectively. The methodology outlined by Ref.^55^ was followed for this purpose. It should be noted that the availability of the IB correction is the reason the temporal analysis is confined to the period from 1900 to the present. Nonetheless, removing the influence of the atmospheric pressure is necessary to reduce the variability of the time series, and ensure compatibility with the altimeter records. After these selection and processing steps, all datasets were resampled to annual mean.

Choosing annual mean time series over monthly ones simplifies the analysis. The sparser data of annual series minimizes noise and emphasizes long-term oscillations. This is especially evident in the EEMD decomposition (detailed below), where the second IMF of the annual series clearly depicts the target process, in contrast to the more ambiguous fourth IMF, found in the monthly series (not shown). Given that the analysis utilizes annual averages, the inherent seasonality present in monthly data is effectively neutralized.

Gap-filling is essential for the EEMD process, so a straightforward linear interpolation was applied for up to three missing consecutive years. Given the interest in capturing oscillatory signals in sea-level data, linear interpolation for gap-filling was chosen to minimize the introduction of artificial information into the series. If gaps were longer, or if segments of the time series were highly discontinuous, those segments or entire series were removed. Following this methodology, the average percentage of data gaps in the analyzed data is approximately 8.8%, suggesting the impact of these gaps can be considered minimal. In cases with extended data gaps, such as Porto Corsini (1922–1933) and Antalya (1978–1984), the time series before and after the gaps were treated as separate entities during the EEMD process. For effective detection of decadal variability within the signal, records at the end of the processing had to span more than 20 continuous years.

The contribution of glacial isostatic adjustment (GIA) on relative sea level (dSea)^56^ has been removed from TG data. The effects of local VLM, attributed to natural processes like sediment compaction and local tectonics, as well as human activities like underground fluid exploitation, often dominate relative sea level discussions. However, no precise method is currently available for extracting this variable from TGs due to the significant variance of VLM in both time and space. The primary tool for addressing VLM is through GNSS stations co-located on TGs^57^. This practice, initiated in the late 1990s, has not yet been universally adopted, making comprehensive corrections for the 20th century challenging. Consequently, this variable was not removed from TG signals in this analysis. Despite this, the focused type of analysis is not significantly impacted, as VLMs, though non-linear, evolve gradually over time. While they do substantially influence the residual in signal decomposition, they exert minimal effect on periodic processes. It’s worth noting that the effect of GIA would primarily influence the residuals as well and not the oscillatory modes. However, given that the correction for GIA is known and available from models, applying it serves to reduce signal variability and effectively remove its contribution from the analysis.

The estimates of the gravitational, rotational, and deformation (GRD) effect on sea level, driven by contemporary mass changes of the Antarctic and Greenland ice sheets, glaciers, and water stored on land, have been retrieved from^58^. However, this product currently covers the period 1900–2018, omitting the last 3–4 years of observations in both TGs and SA. Although this constitutes a brief period, it might be crucial, considering that the most recent observed NIG reversal occurred around 2020. Therefore, the influence of the GRD correction on the quasi-decadal fluctuation has been evaluated by comparing it with the non-corrected series. As shown in Fig. S5, the normalized root mean squared error (NRMSE x 100) of the GRD-corrected and non-corrected series is significantly low across all sub-basins and the entire domain, with an average value of 5.6% for the Mediterranean Sea. This minimal variability should be attributed solely to the magnitude of signals (y-axis), essentially not influencing the occurrence of inflections or the behavior of the oscillation over time (x-axis), which is the main focus of this study. Indeed, this correction primarily affects sea-level trends over longer periods, i.e., the EEMD residuals (Fig. S6). Consequently, to maintain longer observational datasets, the GRD effect on the oscillatory modes is deemed negligible and thus not incorporated into the overall analysis.

In terms of sea-level components, it is recognized that the variability observed in the total sea level is represented by the summation of various drivers^24^. As described above, in this study, contributions from GIA and IB (already excluded from SA) have been removed, and the effects of GRD and VLMs (not influencing SA data) are assessed to have negligible influence on the oscillatory modes. Consequently, the residual variability under observation can solely be attributed to the sterodynamic component. Thus, the analysis primarily focuses on sea-level variability directly tied to density changes due to thermohaline properties (steric component) and the dynamic redistribution of water mass through circulation (dynamic component).

### Satellite altimetry data

The SA data was sourced from the European Seas Gridded Sea Surface Heights product provided by the Copernicus Marine Service (DOI product: https://doi.org/10.48670/moi-00141). As of the time of this analysis, the dataset spans from January 1993 to June 2023, providing daily data fields on a $1/8^{\circ } \times 1/8^{\circ }$ grid covering European seas. The product is generated by the DUACS (Data Unification and Altimeter Combinations System) multi-mission altimeter data processing system, which utilizes data from all available satellites at any given time. Before distribution, all standard and geophysical corrections were applied to the data. These encompass adjustments for orbit drift, sea state bias (reflecting sea roughness), ionospheric and tropospheric path delays (both dry and wet), as well as tidal corrections such as ocean, pole, solid Earth, and loading tides. Moreover, the dataset incorporates the Dynamic Atmospheric Correction^59^, accounting for wind and pressure effects over periods shorter than 20 days and the IB response of the sea for longer duration.

Time series outside the Mediterranean grid, as well as the Black and Marmara seas, were masked out and not considered in the analysis. Following this procedure, a total of 16,404 time series were available for the analysis. Regionalization of sub-basins was computed following^21^. For practical reasons, and due to the limited amount of TGs data, the Western and Tyrrhenian sub-basins were merged and considered as the Western Mediterranean, while the Ionian and the Southern Central sub-basins were merged and regarded as Ionian. Due to data scarcity and the complex sea-level dynamics in the Southern Crete sub-basin, it has not been merged with the adjacent Levantine. Therefore, this sub-basin is excluded from the analysis.

Daily measurements of sea level anomaly were converted into annual means for the analysis, with 2023 excluded to prevent bias since records for that year were only available up to June 7th. SA quantifies geocentric sea-level changes, meaning it measures the sea surface height relative to the reference ellipsoid^24^. These data are not influenced by local VLMs, except for the effect of GIA. Hence, the ICE-6G_C VM5a model (dSea + dRad)^56^ was used to remove the contribution of GIA to geocentric sea-level change. To address the TOPEX-A instrumental drift affecting records from 1993 to 1998, a correction was applied to the altimetry data^60^, which is provided within the product. Although this correction is primarily designed for global mean sea level, it remains the best available estimate for regional or local scales, particularly given the unknown regional variation of the drift.

From the same dataset, the relative vorticity field of the absolute geostrophic component for a specific section of the Ionian Sea was derived. This calculation incorporated both the surface geostrophic eastward and northward seawater velocity components, focusing exclusively on the marine region between 17 and 19.5^∘^E and 37–39^∘^N. The data selection and methodology align with those detailed in^17^. Thus, a time series is obtained related to the surface circulation of the Ionian, specific to the selected area, which indicates cyclonic and anticyclonic phases of the NIG when the vorticity field is positive and negative, respectively.

### EEMD processing

Periodicities within the TGs and SA time series were determined through the use of the Ensemble Empirical Mode Decomposition (EEMD) method^61^. This represents a non-parametric technique that decomposes a signal into a finite set of Intrinsic Mode Functions (IMFs), making it particularly suitable for analyzing non-stationary and non-linear time series, such as sea-level data^62–64^. Given the characteristics of sea level, each IMF could potentially capture a specific dominant oceanic process^62^ at a regional or local scale. These processes may vary across different areas of the globe, according to the local/regional dominant drivers of sea-level change. Thus, when decompositions display similar trends and modes at such spatial scale, common shared processes are suggested^62^. Unlike Principal Component Analysis, which may necessitate a uniform data distribution to function optimally, EEMD can be applied to individual time signals. This is invaluable when dealing with data that are spatially sporadic or non-uniform. Further, EEMD does not operate under the premise of maximizing variance, as Principal Component Analysis does, making it more conducive to handling heterogeneous data sets.

IMFs, serving as a sequence of oscillatory modes from the highest to the lowest frequency, are characterized by their non-constant phase and amplitude, and the method additionally isolates non-oscillatory residual modes^61^. The number of IMFs achieved is strictly linked to the length of the dataset. Generally, the decomposition of annual mean sea-level signals in this study yields five IMFs. These represent (from the highest to the lowest frequency) inter-annual, decadal, and multi-decadal variability (oscillatory modes), in addition to the long-term non-linear and linear trends (residuals). When analyzing shorter time series, the multi-decadal oscillatory mode might not be detected (as for the SA and some TGs data), as the ability to resolve such oscillations in time series requires at least 40 or more years of data. As a result, its influence can remain within the residual signals, thereby ‘contaminating’ them.

A critical aspect of EEMD method rely upon the introduction of white noise in the signal. This incorporation, though initially seeming counter-intuitive, is crucial for enhancing signal decomposition. White noise serves as a reference, facilitating clearer signal scale separation. It minimizes the occurrence of mode aliasing, where components of differing temporal scales may combine. Through the addition of this noise, a stable extraction of IMFs is assured, granting uniformity across scales and a more consistent decomposition. The inclusion of noise sharpens the delineation of closely spaced signal components and diminishes unresolved oscillations in the residuals, ensuring that the majority of oscillations are included within the IMFs. Additionally, by averaging over multiple decompositions, each with a unique noise realization, the ensemble approach ensures that random noise components are neutralized, yielding a more repeatable and reliable representation. For this reason, a 10,000 repetitions EEMD process was applied to any dataset in this analysis, then an ensemble mean of resulted IMFs was built. Subsequently, the individual ensemble means obtained for each TGs were averaged, thereby producing a representative set of IMFs for each Mediterranean sub-basin. To avoid biases related to the uneven distribution of data across the domain, the ensemble mean representative of the whole Mediterranean Sea was produced by averaging the ensemble means from the sub-basins.

The fast Fourier transform was employed to identify the dominant periodicities within the IMFs in both SA and TGs. Due to the uneven distribution of considered TGs across the Mediterranean Sea, and the varying number of observation datasets among sub-basins, a distinct approach was taken. For each sub-basin, a dominant period was calculated along with its associated standard deviation (1$\sigma$). Instead of directly averaging these dominant periods, the Mediterranean’s average period was determined by computing the weighted average of dominant periodicities from all the sub-basins. An associated uncertainty (1$\sigma$), representing the variability of the dominant periodicities across different sub-basins, was also computed using a weighted standard deviation approach. Subsequently, indices of local maxima and minima within each first and second IMFs were detected using a time window of 3 years; this means that each point was compared with the data spanning 1.5 years before and after it to determine whether it was a peak or trough. Subsequently, the occurrences of NIG reversal documented in literature (see the Introduction Section) were integrated into the analysis. This was done to ascertain potential correlations between these occurrences and the primary variability of the IMFs.

### Supplementary Information

Supplementary Information.

## Data Availability

The data used in this study can be freely accessed at the following websites: tide gauges at https://psmsl.org/data/obtaining/ and satellite altimetry at https://data.marine.copernicus.eu/product/SEALEVEL_EUR_PHY_L4_MY_008_068/services. The data generated from this analysis are available at https://zenodo.org/records/10705443. All the analyses and figure production were carried out with Python 3.11.5.
