# Supplementary material for: The potential recording of North Ionian Gyre’s reversals as a decadal signal in sea level during the instrumental period
Source: Sci Rep. 2024 Feb 28;14:4907. doi: 10.1038/s41598-024-55579-4 (PMC10902283; doi:10.1038/s41598-024-55579-4)
Supplement: Supplementary file 1 — Supplementary Information. [file 41598_2024_55579_MOESM1_ESM.pdf]

Supplementary Information

1. Tide gauges selected for this study

Out of all available tide gauge datasets within the Mediterranean Sea, 46 tide gauges were chosen (Table S1). These selected stations met stringent filtering criteria tailored to the objectives of this study: each month required a minimum of 15 days of observations, and each year needed at least nine months of data. Additionally, a maximum gap of three consecutive years was permitted.

sub-basin	PSMSL id	name	lat	lon	time span	notes
Adriatic	154	Trieste	45.64736	13.75847	1900-2021	
	na	Venezia	45.43333	12.33333	1900-2019	From Zanchettin et al., 2021
	na	Porto Corsini	44.49205	12.28273	1900-2015	From Meli and Romagnoli, 2022. The EEMD was computed separately for the periods 1900-1921 and 1934-2015
	353	Bakar	45.3	14.53333	1950-2020	
	352	Split - Gradska Luka	43.5067	16.4417	1954-2018	
	760	Dubrovnik	42.6583	18.0633	1956-2018	
	761	Rovinj	45.0833	13.6283	1956-2018	
	1075	Bar	42.08333	19.08333	1965-1991	
	1859	Zadar	44.1233	15.235	1995-2018	
Aegean	373	Thessaloniki	40.63254	22.93493	1969-2019	
	374	Piraievs	37.93733	23.62671	1969-2022	
	408	Khios	38.37151	26.14119	1969-2015	
	1233	Leros	37.12968	26.84799	1970-2022	
	1234	Siros	37.43997	24.94581	1969-2022	
	1238	Alexandroupolis	40.84414	25.87827	1969-2022	
	1441	Khalkis South	38.46089	23.58946	1977-2022	
	1679	Mentes/Izmir	38.43333	26.71667	1986-2009	
	1680	Bodrum II	37.03333	27.41667	1986-2009	
Ionian	410	Preveza	38.95908	20.75663	1980-2022	
	411	Kalamai	37.02368	22.11584	1969-2019	
	1239	Levkas	38.83454	20.71211	1969-2022	
	1240	Katakolon	37.64482	21.31968	1969-2022	
	1735	Masraxlokk	35.82006	14.53296	1989-2022	
Levantine	253	Port Said	31.25	32.3	1923-1946	
	503	Alexandria	31.21667	29.91667	1944-2006	
	na	Antalya	36.88333	30.7	1936-2008	From Ozturk et al., 2018. Annual means for 1953, 1954, and 1955 have been removed, as mentioned in the paper. The EEMD was computed separately for the periods 1936-1975 and 1986-2008
	1797	Hadera	32.47044	34.86316	1994-2022	
Western	59	Genova	44.4	8.9	1928-2021	Data from the years 2001 to 2014 were sourced and merged from 'Genova II'
	61	Marseille	43.2788	5.35386	1900-2021	

	104	Cagliari	39.2	9.166667	1900-1934	
	105	Napoli (Mandracchio)	40.8429	14.2575	1900-1921	
	106	Civitavecchia	42.05	11.81667	1900-1921	
	107	Palermo	38.13333	13.33333	1900-1921	
	108	Porto Maurizio	43.86667	8.016667	1900-1921	
	488	Tarifa	36.0086	-5.6026	1944-2022	Data from the years 2018 to 2022 were sourced and merged from 'Tarifa 2'
	490	Algeciras	36.12077	-5.43497	1962-2001	
	496	Malaga	36.7127	-4.41546	1962-2022	Data from the years 2013 to 2022 were sourced and merged from 'Malaga II'
	498	Ceuta	35.8924	-5.31589	1944-2018	
	958	Sete	43.3976	3.69911	1992-2021	
	960	Alicante 2	38.33892	-0.48123	1960-2020	
	980	Toulon	43.1129	5.91472	1993-2021	
	981	Gibraltar	36.14826	-5.3649	1962-1982	
	1764	L'Estartit	42.05383	3.206448	1990-2022	
	1811	Barcelona	41.34177	2.1657	1993-2022	
	1813	Valencia	39.44203	-0.31128	1994-2022	
	1892	Palma de Mallorca	39.55238	2.638912	1997-2022	Data from the years 2010 to 2022 were sourced and merged from 'Palma de Mallorca 2'

Table S1. A detailed list of the tide gauges selected for this work.

2. Interannual and multi-decadal sea-level variability

The first IMFs obtained from the decomposition of both TGs and SA data primarily capture the interannual sea-level variability (Fig. S1). For this mode of variability, the regional and sub-basin ensemble mean of the TGs shows a dominant periodicity range between 3 and 4.3 years, with an average of 3.8 ± 1.4 years. The temporal evolution of this signal is notably variable, as expected. The same observation can be made for the first IMF extracted from the SA (regional average periodicity of 3.7 ± 1.2 years), where the spatial variability appears large and chaotic (Fig. S2). Consequently, aligning modes derived from the first IMFs, from both TGs and SA, with NIG reversal episodes is neither straightforward nor feasible in this context. Hence, this study focuses on longer periodicities of sea level, and the reader is referred to e.g. Mohamed et al. (2019), Menna et al. (2022) for a discussion on interannual sea-level variability.

The multi-decadal fluctuation (see IMF 3 in Fig. S6) in signals can only be observed within long TG datasets, e.g., more than 50/60 years of observations, and where smooth non-linearities in VLMs are not overly influential. Generally, a fluctuation within the range of 21-24 years has been detected, varying across the different sub-basins. In shorter time series, this signal is not detectable by the EEMD, which essentially forms an IMF representing the non-linear trend at any specific TG. Indeed, analyzing the multi-decadal (third IMF) or non-linear variability (second to last IMF) of sea level, as well as the residual linear variability (i.e., the last IMF), requires an in-depth examination. This analysis should focus on removing the influence of natural and anthropogenic-induced VLMs and accounting for the influence of mass-driven sea-level change. As a result, multi-decadal signals and residuals are not examined or discussed further in this study.

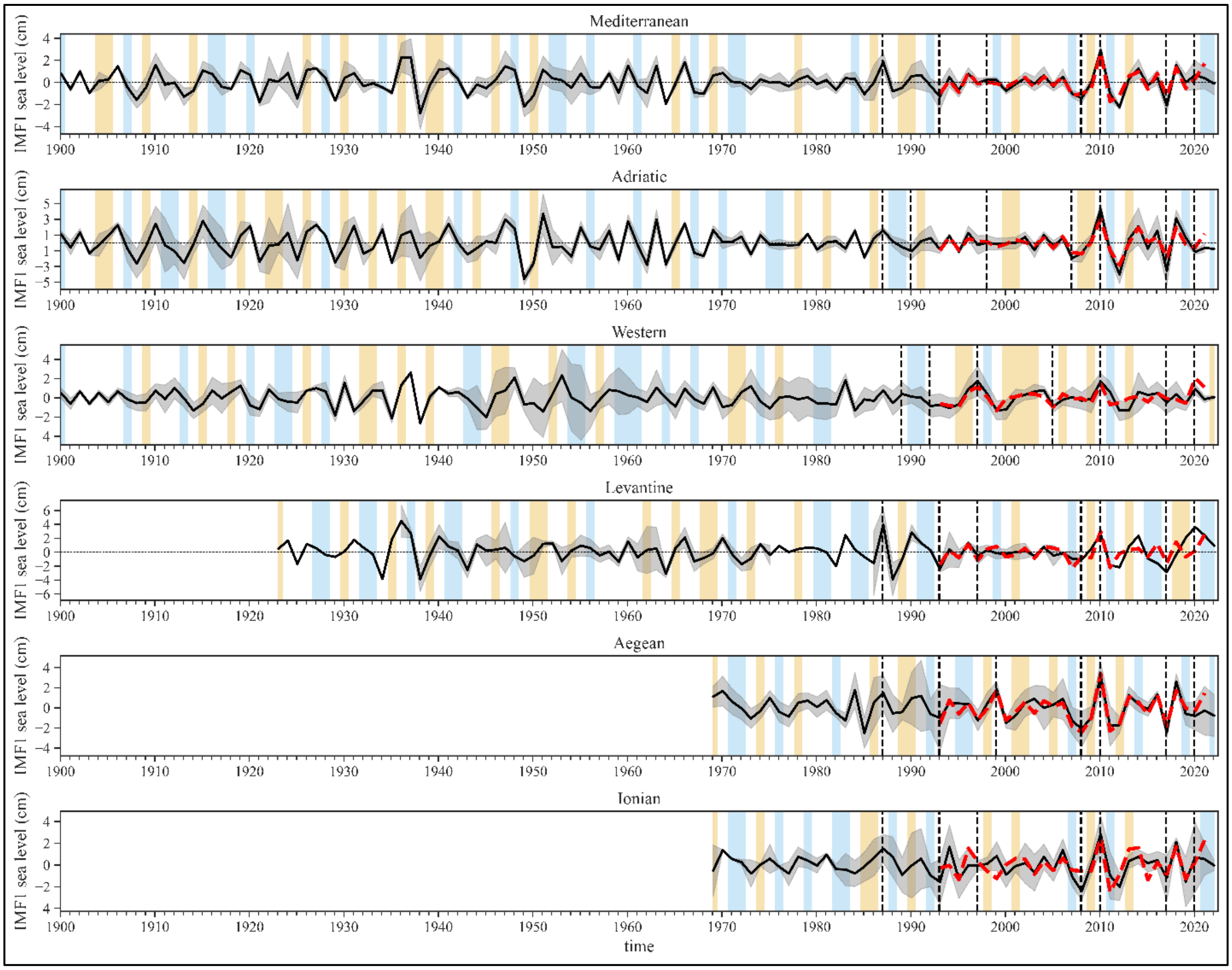

Figure S1. The ensemble mean of the first IMF at the sub–basin scale, computed from TGs, is represented by the solid black line, accompanied by the ± 1σ range (shaded in gray). The red hatched lines depict the basin average mean of the first IMF derived from the altimetry data, whereas the vertical black hatched lines are the same as those used in Fig. 2 (see the main text). The cyan and orange sectors, computed using the same approach as in Fig. 2 from the main text, are intended to symbolize the periods of cyclonic and anticyclonic patterns in the Ionian circulation. However, this reconstruction is shown to be not feasible for the IMF1 sea level.

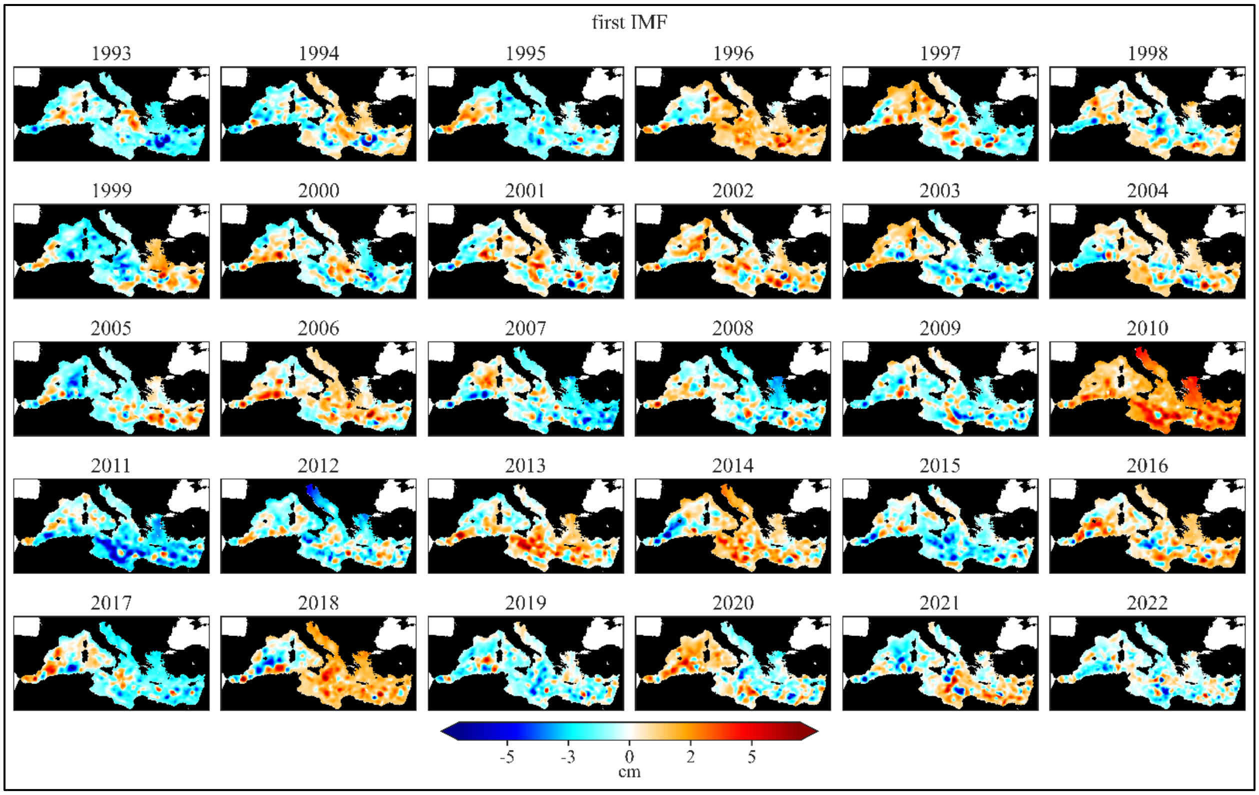

Figure S2. Annual mean value of sea level, associated with the first IMF, extracted from the signal decomposition using EEMD at each altimetry grid point.

3. The sea-surface salinity

Existing literature (refer to the main text for citations) establishes that NIG states, both cyclonic and anticyclonic, induce changes in water mass redistribution and thermohaline properties throughout the basin and its sub-basins. Fluctuations in seawater salinity in the Adriatic Sea have been observed periodically since the early 1900s, and recent studies attribute these changes to the effects of NIG reversal episodes. In this study, the observations align closely with the reconstructed reversals in the Adriatic. Specifically, the observed increases in water salinity (historically termed as 'ingressions') coincide with the commencement of cyclonic phases. As identified in previous studies, during the cyclonic state, the relatively fresher Atlantic Water is diverted directly towards the Levantine Basin after entering the Sicily Channel. This contrasts with its formerly northeastward deflection towards the Adriatic during anticyclonic phases. This results in a relative salinization of the sub-basin and consequently, a change in the halosteric component of sea level. This salinization leads to increased density, causing a localized sea level drop. This effect is further amplified by the reduced contribution of Atlantic Water mass, which is predominantly redistributed to the Levantine during cyclonic phases. Fig. S3 illustrates the variability of sea-surface salinity across the Adriatic Sea from 1987 to 2020. The maps clearly depict a fluctuation in this parameter, with salinity decreasing during anticyclonic phases (1991-1998; 2006-2010; 2017-2020) and increasing during cyclonic phases (1987-1991; 1998-2006; 2010-2017).

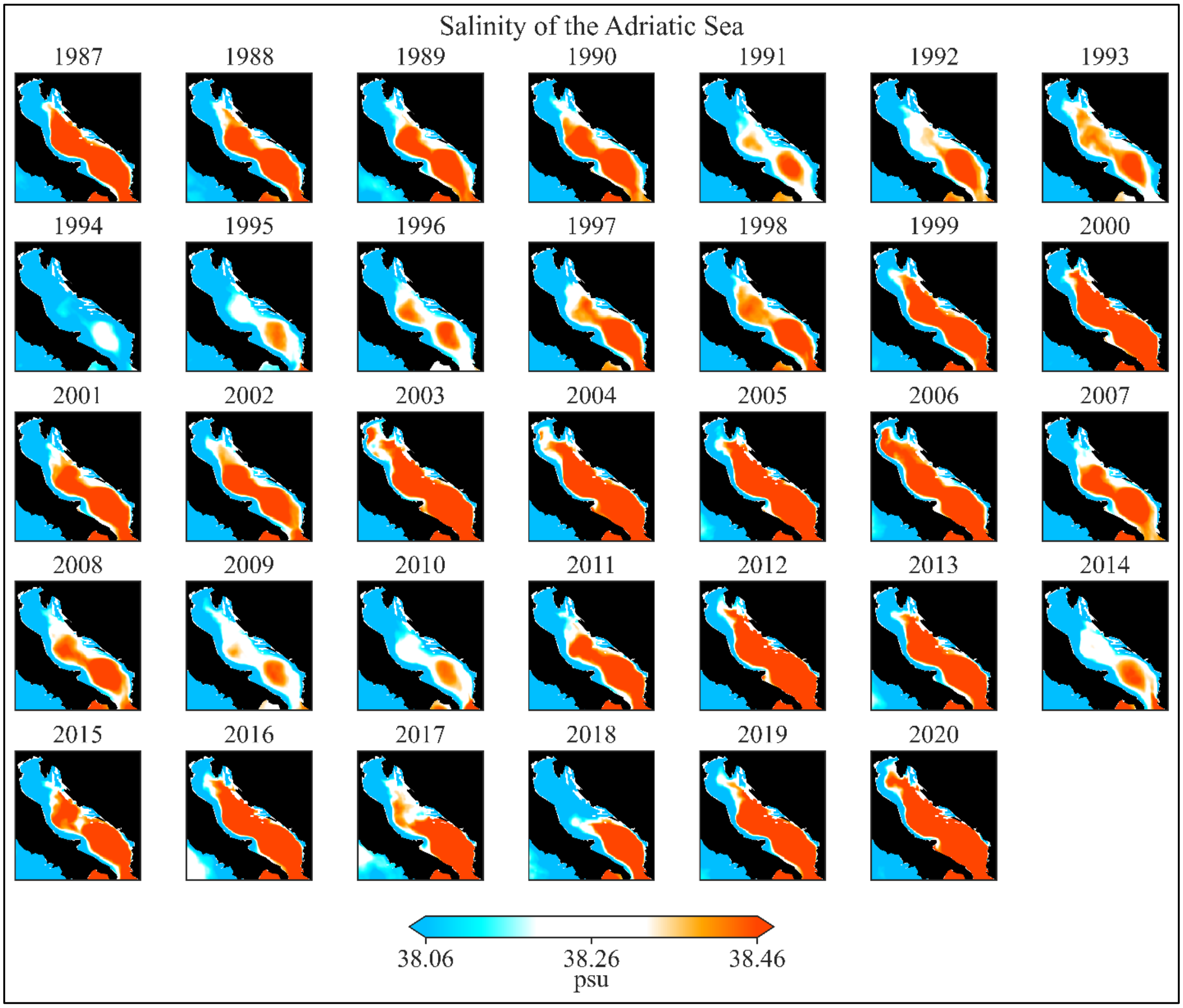

Fig. S3. Sea-surface salinity, corresponding to a depth of 10 meters, across the Adriatic Sea for the years 1987-2020. The colorbar is centered around the average Mediterranean salinity value of 38.26 psu for this period. The data used to produce this figure was retrieved from the Mediterranean Sea Physics Reanalysis, available on the Copernicus Marine Service website (https://doi.org/10.25423/CMCC/MEDSEA_MULTIYEAR_PHY_006_004_E3R1).

4. The North Atlantic Oscillation

The Mediterranean quasi-decadal oscillation achieved from the signal decomposition on TGs has been compared (Fig. S4) with the principal-component (PC)-based indices of the North Atlantic Oscillation (NAO). The NAO PC-based indices serve to gauge the NAO all year round, following the seasonal shifts of the Icelandic low and the Azores high, over the sector 20°-80°N, 90°W-40°E. To reduce noise and enhance comparability, the annual PC-based NAO indices were decomposed (dNAO) using the same approach as for TGs and SA data (refer to the main text). The processing results yielded both the second and third IMF with quasi-decadal periodicities (approximately 8 and 14 years, respectively). Consequently, these two modes were combined for comparison with the Mediterranean-averaged second IMF. In terms of visualization, the Mediterranean signal aligns well with the inverted dNAO, reflecting the well-known opposite sea level response to dNAO

changes. Intriguingly, the signals are in phase during the 1930s-1950s period, with both experiencing a decrease in amplitude. In numerical terms, after accounting for the effective number of samples to remove the autocorrelation, the Kendall tau correlation analysis, based on 10,000 bootstrap repetitions, yields an average tau value of -0.27 and an average p-value of 0.03. This advises that the two signals under analysis are likely significantly correlated. Indeed, the cross-correlation analysis between the two signals reveals significant negative correlations at lags -1 and -2. This suggests that, on a decadal scale, sea level negatively responds to dNAO changes within a period of just 1 or 2 years. Moreover, in terms of linear regression, the magnitude of this variability can be quantified as approximately -0.7 cm of sea level per dNAO unit. Finally, it should be noted that the nature of this correlation must be interpreted with caution. Dedicated studies, involving thorough analyses, are essential to accurately quantify and understand this correlation, as well as its potential link with NIG reversal occurrences.

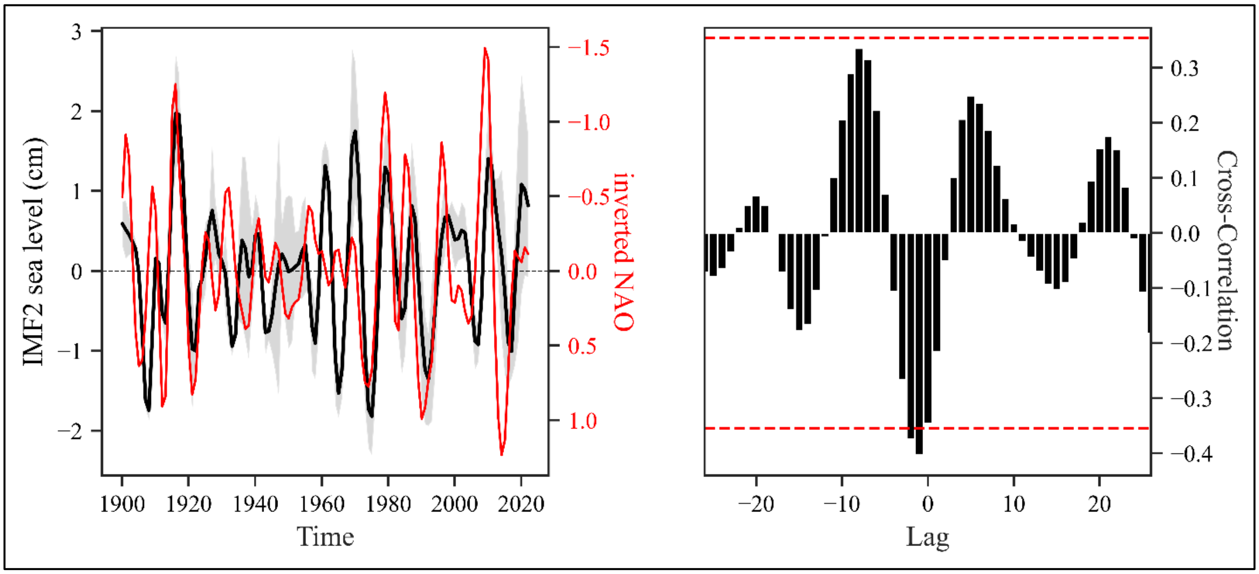

Fig. S4. (Left panel) The Mediterranean-averaged second IMF (black line) with its related 1σ interval (gray shaded area), along with the second + third IMF signal (red line) decomposed from the annual NAO PC-based data (https://climatedataguide.ucar.edu/climate-data/hurrell-north-atlantic-oscillation-nao-index-pc-based). (Right panel) The cross-correlation between the two signals, showing significance at the 99th percentile CI for lags -1 and -2.

5. The sea-level components

The sea-level variability observed at a specific location represents the change in a particular component (or multiple components simultaneously) within what is termed the total sea level ($\eta total$). The $\eta total$ term is essentially the sum of all components, also considered drivers:

$$\eta total = \Sigma drivers = \eta sterodynamic + \eta GRD + \eta GIA + \eta VLMs + \eta IB$$

where $\eta sterodynamic$ represents the variability induced by changes in temperature and salinity (steric term) and mass redistribution through circulation (dynamic term), ηGRD the effect of contemporary mass exchanges between the Antarctic and Greenland ice

sheets, glaciers, water stored on land and the Ocean, ηGIA the glacial isostatic adjustment, i.e., the ongoing movement of land once loaded by ice-age glaciers, $\eta VLMs$ the contribution of all vertical movements at a specific location induced by local tectonics, sediment compaction and underground fluids exploitation (this term does not affect altimetry), and ηIB is the inverse barometer effect which accounts for the contribution of atmospheric loading on sea level. It is recognized in literature that shifts in the NIG state result in changes to both thermohaline properties and water mass distribution, essentially altering the sterodynamic component. Given that this study centers on the sea-level variability prompted by NIG reversal episodes, it is crucial to filter out all sea-level components except for the sterodynamic component. As described in the main text, the datasets used in this study have been corrected for both IB and GIA terms. Furthermore, it is assumed that, although VLMs on tide gauges can be non-linear in time, they do not affect oscillatory modes; thus, the influence of this component is considered negligible. The effect induced by GRD is therefore explored, finding that, in this case as well, the oscillatory modes are very minimally impacted with NRMSE values ranging from 2.6% to 8.8%, which vary across sub-basins (Fig. S5). This component, similar to the VLM, strongly affects only the residuals, as observed in the full set of Trieste IMFs, for example (see Fig. S6). Thus, the GRD component can also be considered negligible when examining oscillatory modes. In this context, the signal being analyzed can be attributed solely to variations related to the sterodynamic component.

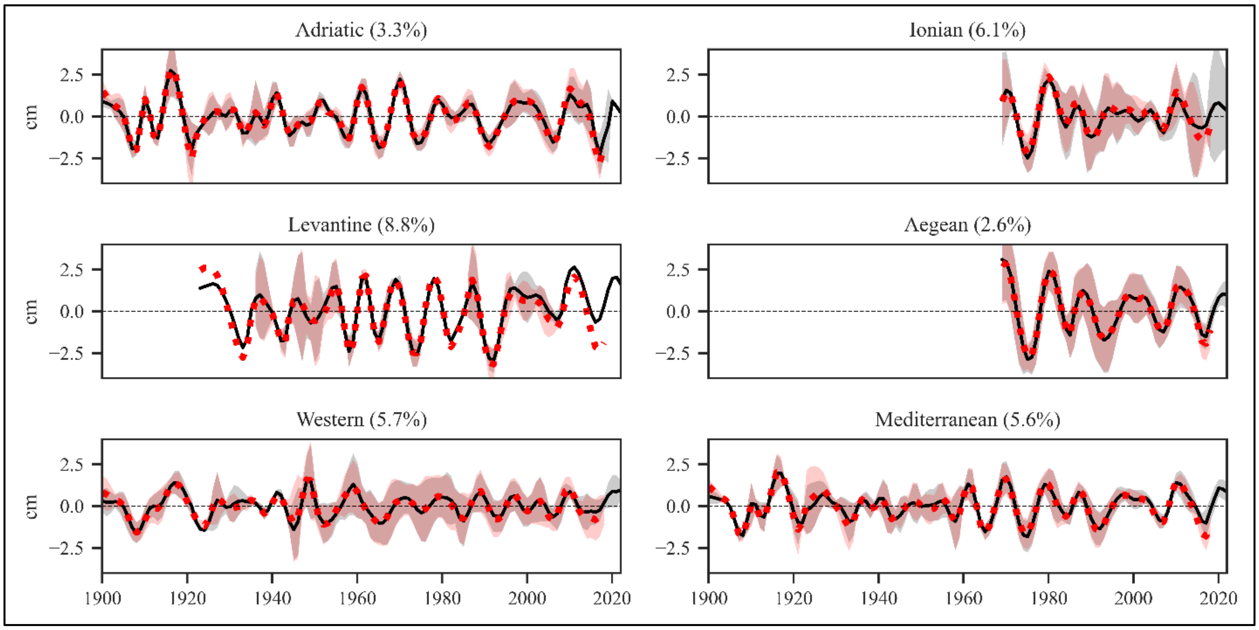

Fig. S5. The ensemble mean of the second IMF sea level at the sub-basin scale, along with its related 1σ values (shaded areas), is shown both corrected (red hatched line) and non-corrected (black solid line) for GRD. The difference between the corrected and non-corrected time series is expressed in terms of NRMSE x 100, as indicated in parentheses.

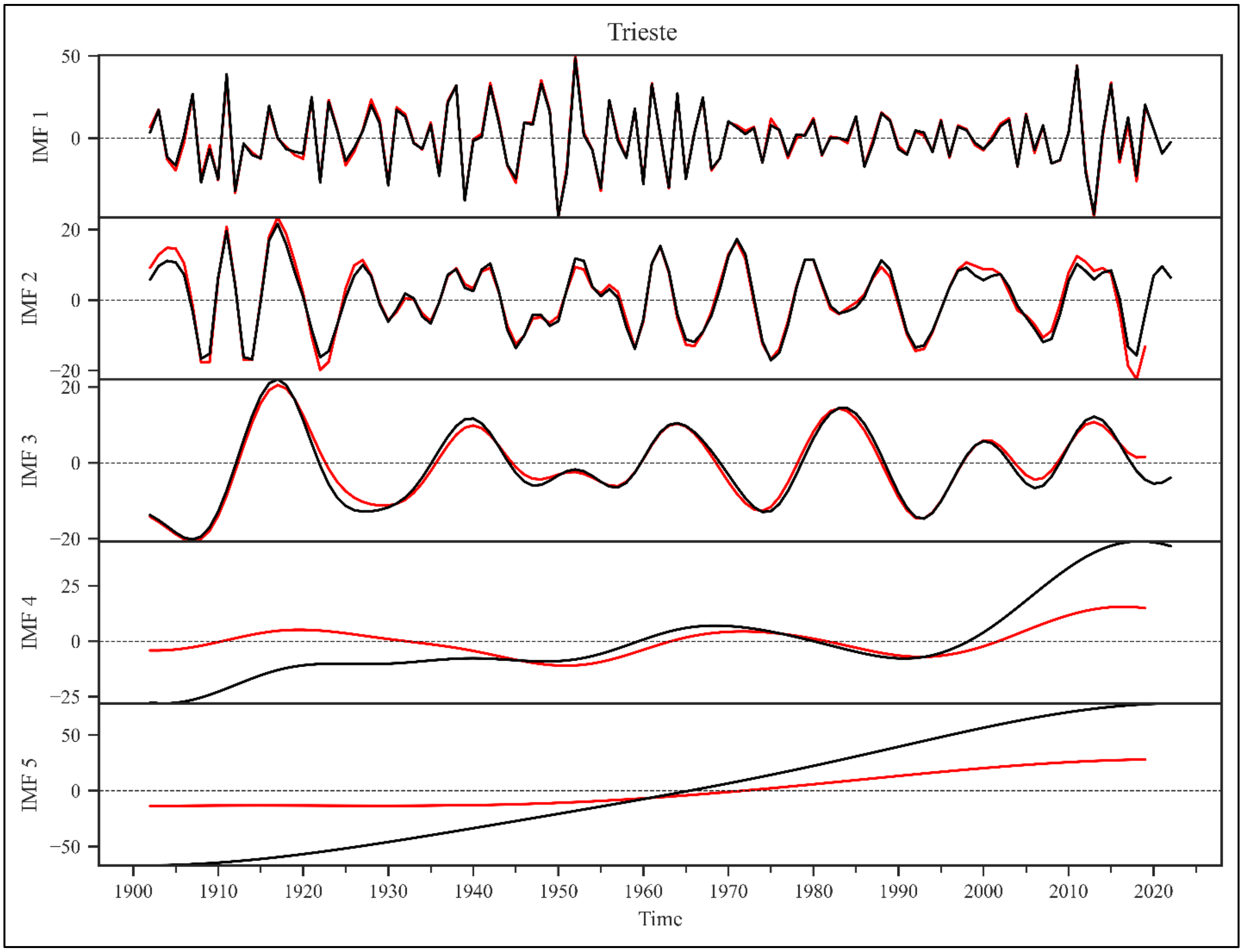

Fig. S6. The five IMFs extracted from the Trieste TG signal (top panel) using the EEMD method. Displayed are the interannual (IMF 1), decadal (IMF 2), multidecadal (IMF 3), non-linear residual (IMF 4), and linear residual components (IMF 5). Both the GRD-corrected (red solid line) and non-corrected versions (black solid line) are shown. Values on the y-axis represent sea-level changes in mm.

6. Observations of quasi-decadal sea-level changes within the Ionian Sea

As stated in the main text, the reconstructed signal for the second mode in the Ionian Sea level, averaged from five TGs (Fig. S7), cannot be considered reliable as this sub-basin is underrepresented by in-situ data and represents the epicenter of the reversal. The latter, essentially occurring in the open ocean, is not adequately captured by TGs, which are attached to the land, but they are still able to provide some interesting information. Since the reversal signal might also propagate towards certain land areas (see Fig. 3 in the main text), its influence on the nearest TGs cannot be excluded. If the quasi-decadal signal, decomposed from the sterodynamic sea level, were not related to reversals, a similar signal among TGs within the basin and with the adjacent sub-basin would be expected. Conversely, as shown in Fig. S7, this signal is often out of phase among the five TGs available, also providing indications of opposite inflections in sea level, both in comparison to signals from other stations and especially to signals from TGs in adjacent sub-basins (Fig. 2 in the main text). This can be explained in terms of consequences related to the breathing oscillation pattern, linked to NIG reversals, which might actually influence some coastal areas

around the north Ionian Sea by propagating towards the land and also moving in time and space. This consideration can strengthen the hypothesis of a direct link between the quasi-decadal oscillatory signal hidden in the Mediterranean sea level and NIG reversal occurrences. Additionally, it should also be considered that the averaged interannual sea-level signal over the Ionian, despite representing a general chaotic pattern, is much more coherent in time among the five stations (Fig. S1). Indeed, the interannual signal does not seem to be related to NIG reversals.

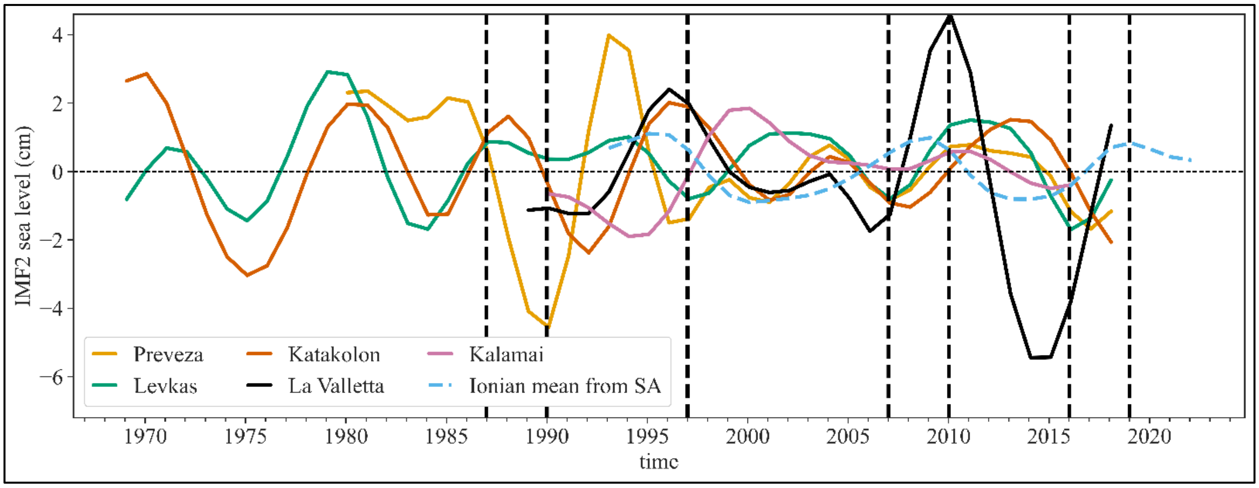

Fig. S7. The second IMF signal extracted from the SA and five TGs considered for the Ionian Sea. The vertical black hatched lines represent the occurrence of known reversals from the literature (see the main text).

References

Escudier, R., Clementi, E., Cipollone, A., Pistoia, J., Drudi, M., Grandi, A., Lyubartsev, V., Lecci, R., Aydogdu, A., Delrosso, D., Omar, M., Masina, S., Coppini, G., Pinardi, N. A High Resolution Reanalysis for the Mediterranean Sea. *Frontiers in Earth Science*. **9**, https://doi.org/10.3389/feart.2021.702285 (2021).

Hurrell, J., van Loon, H. Decadal variations in climate associated with the north Atlantic oscillation. *Clim. Chang*. **36**, https://doi.org/10.1023/A:1005314315270, (1997).

Hurrell, J. NAO Index Data provided by the Climate Analysis Section, NCAR, Boulder, USA. Updated regularly. Accessed 14 November 2023. (2003).

Menna, M., Gačić, M., Martellucci, R., Notarstefano, G., Fedele, G., Mauri, E., Gerin, R., Poulain, P.-M. Climatic, Decadal, and Interannual Variability in the Upper Layer of the Mediterranean Sea Using Remotely Sensed and In-Situ Data. *Remote Sensing*. **14**, https://doi.org/10.3390/rs14061322 (2022).

Mohamed, B., Abdallah, A.M., El-Din, K.A., Nagy, H., Shaltout, M. Inter-Annual Variability and Trends of Sea Level and Sea Surface Temperature in the Mediterranean Sea over the Last 25 Years. *Pure and Applied Geophysics*. **176**, https://doi.org/10.1007/s00024-019-02156-w (2019).
